# Solstice, selection, and synchrony of seed masting

**DOI:** 10.1073/pnas.2515264122

**Published:** 2025-08-18

**Authors:** Michał Bogdziewicz, Andrew Hacket-Pain, Dave Kelly, Jakub Szymkowiak, Jessie Foest, Valentin Journé

**Affiliations:** ^a^Forest Biology Center, Institute of Environmental Biology, Faculty of Biology, Adam Mickiewicz University, Poznan 61-614, Poland; ^b^Department of Geography and Planning, School of Environmental Sciences, University of Liverpool, Liverpool L69 7ZT, United Kingdom; ^c^School of Biological Sciences, University of Canterbury, Christchurch 8140, New Zealand; ^d^Population Ecology Research Unit, Institute of Environmental Biology, Faculty of Biology, Adam Mickiewicz University, Poznan 61-614, Poland; ^e^Department of Biology, Faculty of Science, Kyushu University, Fukuoka 819-0395, Japan

Two recent papers showed that in temperate trees, the summer solstice marks a reversal in temperature effects on leaf senescence (1) and initiates temperature cue sensing for synchronous mast seeding (2). Meersch and Wolkovich (3) propose that for both growth and reproduction, the solstice may serve not as a direct cue but as a proxy for peak thermal predictability. They emphasize that the key question is the fitness consequences of cue use. However, the fitness consequences of a solstice-linked signal differ fundamentally between mast seeding and typical growth processes.

In wood and leaf phenology, the solstice, or a nearby thermal optimum, has been proposed as a direct cue for physiological change (1, 3, 4). For most growth-related traits, fitness depends on within-plant optimization to maximize resource gain, favoring cues that allow local adaptation. This makes the solstice a suboptimal cue (3), being insensitive to a plant’s resource status or the site environmental conditions. Van der Meersch and Wolkovich illustrate this with the trade-off between extending growth and avoiding late frost damage. Supporting this, Zohner et al. (1) showed that the mean date when temperature effects on leaf senescence reversed, once near the solstice, has advanced over the past four decades.

**Fig. 1.**
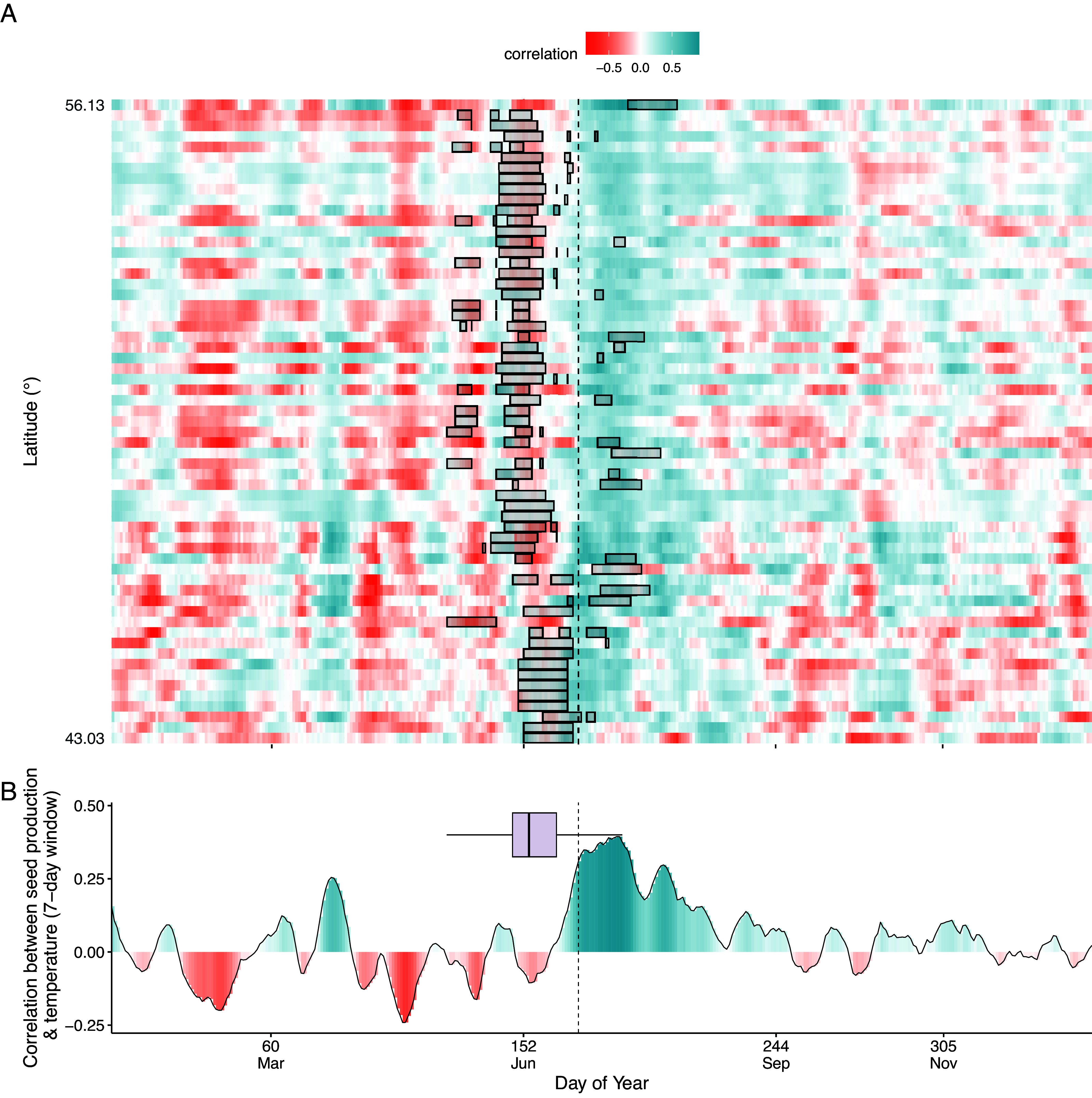
At sites throughout Europe, European beech starts responding to temperature cues synchronously at the summer solstice, even though sites vary in the times of thermal optima. The graphs show correlations from January to December of the year before seedfall (which ripens and disperses the following September). (*A*) Moving window Spearman correlations between temperature and masting across 61 sites distributed over the European beech range. The size of the temperature window is 7 d, with a 1-d step. The black dashed line indicates the summer solstice (21 June). Each row is a site, sorted by latitude (43.03^°^ to 56.13^°^), and data are described in ref. 2. The black rectangle at each row highlights the thermal optimum (the top 10% of days when the trade-off between environmental predictability and growth potential is maximized) for each site, as described in ref. 3. (*B*) Mean rolling Spearman correlation (red line) between temperature and masting averaged across all 61 sites. Correlations are coded blue for positive, and red for negative. Optimality (black boxplot) precedes the solstice on average, but varies widely between sites.

In contrast, synchrony among individuals is essential for maximizing fitness under mast seeding. In high-seeding years, synchrony enhances pollination efficiency and predator satiation (5). Thus, it is a benefit, not a drawback, that the solstice is unaffected by plant-level or site environmental conditions. The solstice does not influence reproduction directly (e.g., via photosynthesis) or serve as a cue to initiate flowering. Instead, it signals all individuals across the species’ range to begin sensing temperature cues for flowering simultaneously (2). This synchronized cue onset enables the remarkable spatial synchrony in seed production observed in European beech (6). In contrast, the “optimal date” proposed by Van der Meersch and Wolkovich, being site-dependent (figure 2 in their study), would reduce synchrony. Beech populations across Europe begin temperature sensing at the solstice, not at variable local optima (Fig. 1).

Clarifying these distinctions is crucial as climate change continues to disrupt phenological systems. Van der Meersch and Wolkovich caution that “using a fixed date like the summer solstice as a cue... could limit plasticity in how plants respond across their ranges, which span very different climates.” Yet this very lack of plasticity is what enables strong spatial synchrony in masting species. The concern, however, lies not in spatial variation but in temporal change. For beech, the fixed solstice cue combined with rising summer temperatures may underlie recent declines in viable seed production and radial growth, driven by a breakdown in masting (7, 8).
